# The Flowing Tide of Christmas Charity

**Published:** 1894-12-22

**Authors:** 


### The Hospital

AN INSTITUTIONAL JOURNAL OF

The Medical Sciences and Hospital Administration,

WITH

Vol. XVII.—No. 430.] AN EXTRA NURSING SUPPLEMENT. [Dec. 22, 1894.

### A Flowing Tide of Christmas Charity.

The strength of the appeal which hospitals make at Christmas to all classes of people who have money to give lies in the merits of the case. The arguments employed may seem feeble, and the attempt to awaken sympathy may fail for lack of skill and happy phrase on the part of the writer. But the care and cure of the sick and injured in our hospitals is in our times so well done, and the work itself is so entirely noble and indispensable, that neither feeble arguments nor want of capacity to touch the deeper springs of feeling in the reader can diminish by a feather's weight the force of an appeal which is at all times practically irresistible. The writer of a Christmas article on hospitals and their work may therefore enter upon a familiar and oft-repeated duty with a good courage.

There is another and an almost equally inspiring side to this Christmas obligation. The English people are, on the whole, a well-meaning people, a very well-meaning people, let the foreigner say what he will. Do but convince them that a thing is worth doing, and that nobody else can or will do that particular thing at the moment so well as themselves, and they will metaphorically take off their coats, and set to work with a will, neither boasting nor resting until the thing is done. Now the hospitals and their suffering inmates need a certain amount of additional help at Christmas time. There is nobody but ourselves, the Christmas-loving Englishmen and Englishwomen, to do it. We have taught them to expect a generous flow of money into their coffers at this period. Not that the money is spent upon Christmas festivities or frivolities, very far from it. The full coffers of Christmas time go to meet the plain daily necessities of those less generous periods when men are too busy to think of hospitals, and when, therefore, the hospital wards would be very distressful places indeed if there were not a little of the genial Christmas hoard left to stop the gap. But this flowing tide of Christmas gifts, we say, the public has taught hospitals and their inmates to look for every year. It would be a failure to meet an implied obligation, an obligation of honour, since there is no definite agreement, written or otherwise, if we were to fail the hospitals this Christmas. To maintain our hospitals is a thing which honour, humanity, and that deep John Bull feeling which lies more or less hidden in all our hearts, bids us to rise up and do. And we must do it. Not less, but more, if possible, must we give this year than ever we have given before.

Let us remind ourselves of some facts and considerations. Bad times may diminish wealth, but they do not lessen suffering or put a bridle on disease. Perhaps, indeed, they foster both disease and suffering by tightening the grip of poverty upon the throat of the poor. The hospitals, therefore, cannot bend to the pressure of the times as private persons can, by diminishing their activities and their expenditure. On the contrary, bad times are just the periods when hospital doors must be thrown most widely open. Poverty itself is a breeder of diseases; and the wholesome antiseptic of hospital activity must be strengthened and concentrated for those periods. Moreover, our hospitals, notwithstanding that they are such noble British institutions, and that we are all so proud of them, have not as yet taxed the resources of many of us very severely. A few people, it is true, have bestowed money and energy upon them with a generosity truly magnificent. But the many, even among the prosperous classes, have never yet got beyond the one poor annual guinea, with, perhaps, an added half-crown for the Hospital Sunday collection. The guinea, compared with the object, is not much. Very few of us are proud of it in our heart of hearts. We should be able to look ourselves in the face with more satisfaction if the guinea were in many cases five, or ten, or even twenty. A great charitable object is not to be kept great except by great sympathy of feeling and great generosity of practical help. It is not natural for Englishmen to be pharisaical. Let us not be pharisaical and self-deluding in this matter of gifts to hospitals.

The readers of this journal have constantly had put before them, from almost every point of view, the magnitude, the enormous mass, of the work done for the sick and injured, free of cost, in this country. It is quite certain that very few of them realise in the faintest degree what that mass means. If they did, they would feel that the small and occasional efforts at hospital support made by the majority of Englishmen are little better than triflings with a great subject. It is the few who hitherto have undertaken the Herculean task of keeping our great hospitals in the forefront of the science and benevolence of this great century. Of the many, some have sympathised and helped a little; others have neither helped nor sympathised at all. But we once more invite the many to give a little time to the investigation of the subject. In London alone there are no fewer than one hundred and seventy-five hospitals and dispensaries, all of which are, to all intents and purposes, free. Thirty-two of these are all-round "general" hospitals, and some of them are famous for their magnitude and the splendour of their work from north to south and from the rising to the setting of the sun. Fifty-two are devoted to the various medical and surgical specialisms; and of some of them it may be said that they are the good angels of modern science, the tread of whose feet has caused health and life to spring up everywhere like beautiful and perennial flowers from barren ground. These various London hospitals provide more than nine thousand beds for in-patients, which are occupied by more than ninety thousand different individuals every year. Of out-patients the name is "legion," and in London alone they are counted almost by millions annually. The amount of money spent every year on these one hundred and seventy-five hospitals is prodigious; and yet when we consider the vast extent and the nature of the work done we cannot but be amazed that the cost of it all is not ten times greater than it is. To destroy an army of ninety thousand men would cost millions, and perhaps even tens of millions. The London hospitals cure and bring back to life every year an army of more than ninety thousand who are dangerously sick, and at a cost of little more than the half of one million. This is a fact which forces itself upon the least reflective mind.

Kindly thoughts are bred in kindly hearts. Those whose Christmas season is this year to be spent in hospitals are of our own blood; they were born in the same land; they live under the same laws; they are defenders of the same flag; they are baptised into the same faith; at death the same solemn rights will commit their bodies to the ground. Let us be tender to them; let us be generous. The heart that gives tenderly and generously gives not once nor twice, but multiplies its gifts by more than ten thousand times ten thousand.

